# Effect of the Sodium Silicate Inhibitor on the Corrosion Protection of AZ31 Magnesium Alloy

**DOI:** 10.3390/ma17225533

**Published:** 2024-11-13

**Authors:** Jiawei Li, Tao Lai, Yang Chen, Hong Yan, Honggun Song, Chao Luo, Zhi Hu

**Affiliations:** 1 School of Advanced Manufacturing, Nanchang University, Nanchang 330031, China; lijiawei_0518@163.com (J.L.); laitao_6666@126.com (T.L.); chenyang@ncu.edu.cn (Y.C.); yanhong_wh@163.com (H.Y.); songhonggun@ncu.edu.cn (H.S.); luochao@ncu.edu.cn (C.L.); 2 Institute for Advanced Study, Nanchang University, Nanchang 330031, China

**Keywords:** magnesium alloy, inhibitor, adsorption, corrosion resistance, molecular dynamics

## Abstract

The effect of the sodium silicate inhibitor on the corrosion protection of the AZ31 magnesium alloy at room temperature was investigated. The results of electrochemical measurement and weight loss experiments showed that incorporating the sodium silicate significantly enhanced the anti-corrosion property of the AZ31 alloy. When the alloy was immersed in the corrosive solution with the 0.1 M sodium silicate, the corrosion rate of the AZ31 alloy declined to 0.014 mm·y^−1^, and the inhibition efficiency reached 99.1%. The observation of the corrosion morphology indicated that the magnesium silicate precipitated to cover the corroded area with a thickness of 105 μm, while the silicate ion adsorbed on the uncorroded area. The calculation results of the adsorption energy based on the molecular dynamics indicated that the physical adsorption occurred when the samples were immersed in a sodium silicate solution. Combined with the schematic diagram, the protective mechanism of the adsorption and precipitation after the addition of the sodium silicate inhibitor was investigated.

## 1. Introduction

The magnesium (Mg) alloy is a light material with high specific strength, machining properties, and anti-electromagnetic interference properties [1,2,3,4,5,6,7]. Therefore, the Mg alloy can be widely used in the automotive, electronics, aerospace, and military industries [8]. However, the occurrence of micro-galvanic corrosion is facilitated by the significant potential difference between the phases in the Mg alloy, leading to suboptimal corrosion resistance [9]. Therefore, improving the anti-corrosion properties in Mg alloys has generated significant research interest [10,11,12].

At present, the application of corrosion inhibitors requires high corrosion inhibition efficiency and is environmentally friendly [13]. Sodium silicate, an inorganic and natural ground element, has been widely used in the chemical industry. Among the sodium silicate applications, a “green” corrosion inhibitor has been widely reported [14,15]. Cai et al. [16] found that adding silicate during the microarc oxidation process can form a Si-film, which significantly enhances the anti-corrosion property of AZ91 Mg alloys. Liang et al. [17] reported that the silicate coating showed better corrosion resistance than a phosphate coating in the plasma electrolytic oxidation process of the AM50 Mg alloy. Gao et al. [13] indicated that sodium silicate could restrain the corrosion in the AZ91 Mg alloy, and sodium silicate’s optimum concentration and pH value range were investigated. Moreover, numerous pieces of literature [18,19] have shown that silicate leads to the formation of a precipitate layer by chemically reacting with Mg^2+^, which protects Mg alloys from corrosion. However, this explanation of the inhibition mechanism of sodium silicate is not detailed enough, and further research is needed.

In addition to precipitation, the adsorption capacity of molecules is also an important property of corrosion inhibitors. It has been widely known that sodium silicate protects minerals by selectively adsorbing on the surfaces [20,21]. Danielle R et al. [22,23] indicated that the adsorption of the sodium silicate onto the mineral surface can depress its charge. Wang et al. [24] reported that sodium silicate reacted with Mg to adsorb on the forsterite surface and form a protective layer to enhance the corrosion resistance. Azizi et al. [25] conducted a study on the adsorption of sodium silicate using density functional theory. They indicated that the formation of the bonding between the silicate and the surface of kaolinite was attributed to the hybridization of hydrogen and oxygen atoms. Moreover, Hao et al. [26] simulated the adsorption of sodium silicate at the solid–liquid interface of quartz using molecular dynamics methods. They concluded that the components adsorbed on the quartz were mainly SiO(OH)_3_^−^ and Si(OH)_4_. Through simulation calculations, these scholars explained the protective effect of sodium silicate on minerals from adsorption theory and reached a series of conclusions. However, the relationship between precipitation and sodium silicate adsorption has not been clarified.

In this paper, the anti-corrosion property of the AZ31 Mg alloy with different concentrations of sodium silicate addition was investigated via experiments and molecular dynamics calculation, which mainly focused on the protective mechanism of sodium silicate at room temperature [27,28,29].

## 2. Experimental Methods

### 2.1. Materials’ Preparation

The compositions of AZ31 Mg alloy were carried out through inductively coupled plasma atom emission spectrometry (ICP-AES Optima 5300DV, Waltham, MA, USA) measurements, as shown in Table 1. All samples were cut into circular plates measuring 18 mm in diameter and 5 mm in thickness. Then, samples were polished from 100^#^ to 2000^#^ using silicon carbide sandpaper with deionized water. The SiO_2_/Na_2_O ratio of sodium silicate was 1.03 [30].

### 2.2. Electrochemical Measurements

Electrochemical measurements were conducted on a Princeton P4000 electrochemical workstation with a three-electrode system. The working electrode contacted the samples in a 1 cm^2^ area. The reference electrode used in the experiment was a saturated calomel electrode (SCE), while the auxiliary electrode employed was a platinum plate. The samples were tested in 3.5 wt. % NaCl containing different concentrations of sodium silicate for 20 min at 25 °C. The polarization curves were obtained at a scanning rate of 1 mV·s^−1^ in the range of ±250 mV vs. SCE. The electrochemical impedance spectroscopy (EIS) frequency ranged from 10^5^ to 10^−1^ Hz, and the perturbation amplitude was five mV relative to OCP. Versa Studio and ZView software (Princeton P4000 and version 2.0) analyzed the polarization curves and EIS data. The corrosion inhibition efficiency (*η*) was calculated by [31]

(1) $$\eta =\frac{i_{blank}-i_{x}}{i_{blank}}$$

where *i_blank_* and *i_x_* are the corrosion current density (*i_corr_*) of blank concentration of sodium silicate and x M sodium silicate addition (x = 0.05, 0.08, 0.10, and 0.15), respectively. The corrosion rate (*CR_I_*) that was estimated from *i_corr_* was calculated by [32]

(2) $${CR}_{I}=22.85i_{corr}$$

### 2.3. Immersion Experiment

The samples were submerged for 24 h in 3.5 wt. % NaCl solution with varying contents of sodium silicate. All measurements were repeated 3 times at 25 °C to lessen the error. Hydrogen was collected through an inverted funnel and acid burette. After immersion, each sample was immersed in a mixture solution of 10 g·L^−1^ CrO_3_ and 0.5 g·L^−1^ AgNO_3_ to eliminate the corrosion products. Weight loss rate was achieved by the difference in quality between before and after soaking. The mean hydrogen evolution rate and mass loss rate were calculated by [33]

(3) $$V_{\left(\mathrm{mL}Â\cdot {\mathrm{cm}}^{-2}Â\cdot h^{-1}\right)}=\frac{H_{i}\left(\mathrm{mL}\right)-H_{m}\left(\mathrm{mL}\right)}{S\left({\mathrm{cm}}^{2}\right)\times t_{m}\left(h\right)}$$

(4) $$V_{\left(mgÂ\cdot cm^{-2}Â\cdot d^{-1}\right)}=\frac{M_{i}\left(mg\right)-M_{f}(mg)}{S(cm^{2})\times t(d)}$$

where *H_i_* and *H_m_* are the acid burette’s level at the initial and m hour (m = 2, 4, 6, 8, 18, 20, 22, and 24), *M_i_* and *M_f_* are the initial and final weight, *S* is the immersion area, *t* (1 day) is the total immersion time, and *t_m_* is the immersion time after m hours. The corrosion rate (*CR_W_* and *CR_H_*) that was estimated from the weight loss rate and hydrogen evolution rate of 24 h immersion was calculated by [32]

(5) $${CR}_{W}=2.1V_{\left(mgÂ\cdot cm^{-2}Â\cdot d^{-1}\right)}$$

(6) $${CR}_{H}=2.279V_{\left(mLÂ\cdot cm^{-2}Â\cdot d^{-1}\right)}$$

### 2.4. Corrosion Morphology Analysis

For microstructure observation, the sample was polished with alcohol using a 5–0.5 um diamond pasting. After immersing for 24 h, clean the surface with anhydrous ethanol and blow dry the specimen, which is characterized by scanning electron microscopy (SEM, Quanta 200FEG, Hillsboro, OR, USA). The chemical states of the corrosion product elements present on the alloy surface were analyzed using X-ray photoelectron spectroscopy (XPS) with monochromatic Al Kα radiation (1486.6 eV). The C1s peak (284.8 eV) standardized the precise binding energy, and the Thermo Avantage software (version 5.9931) was used to fit the XPS results.

### 2.5. Calculation Details

After importing the primitive crystal structure of Mg from the Materials Studio crystal database, the Mg (0 0 0 1) surface was built for five layers. The Mg (0 0 0 1) presented the lowest surface energy and was adequate for energy convergency. The sodium silicate and water molecules were generated using the Visualizer module and then packed into the solution box using the Amorphous Cell module. As the concentration of sodium silicate was 0.1 M, the solution system was calculated to require the addition of 1000 water molecules and two sodium silicate molecules. The solution box’s a-axis and b-axis orientation lattice lengths were set to 32.1 Å and 55.6 Å, respectively, matching the Mg surface’s. A vacuum layer of 30 Å was built above the water molecule layer to avoid the impact of periodic action. Subsequently, molecular dynamics simulation was performed using the Forcite module, with the interactions and potential energy among the molecules calculated via the CVFF forcefield. Our calculations were performed using the Andersen thermostat function in conjunction with an established (NVT) ensemble at a constant temperature of 298.0 K with a timestep of 1.0 fs. A simulation time of 200 ps was utilized to guarantee that the temperature and energy reached an equilibrium state.

## 3. Results

### 3.1. Electrochemical Experiments

**Figure 1:**
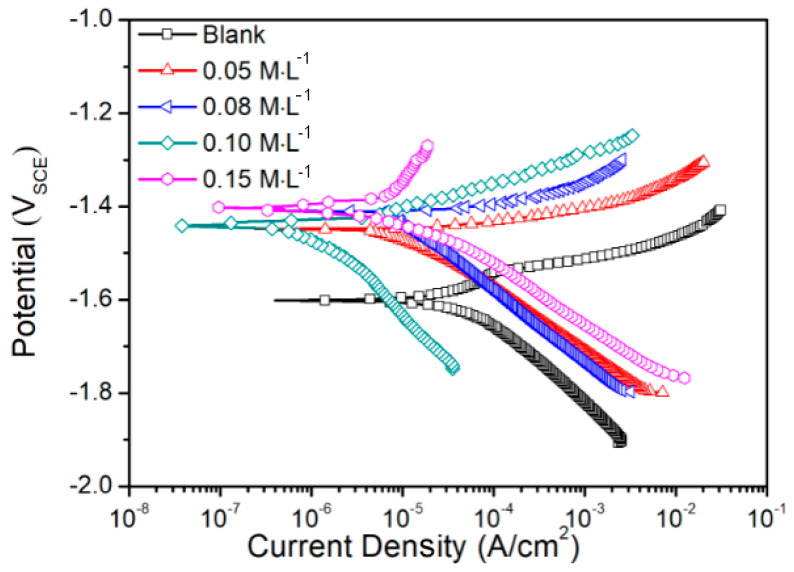
Polarization curves of AZ31 Mg alloy measured in the solution containing 3.5 wt. % NaCl and x M sodium silicate (x = 0, 0.05, 0.08, 0.10, and 0.15).

The polarization curves were measured in a solution comprising 3.5 wt. % NaCl and x M sodium silicate (x = 0, 0.05, 0.08, 0.10, and 0.15), as shown in Figure 1, and the fitting results are listed in Table 2. For the blank concentration of sodium silicate, the *E_corr_* and *i_corr_* values were −1.60 V and 62.1 μA·cm^−2^, respectively. In the solution with a 0.05 M sodium silicate addition, the *E_corr_* value shifted positively to −1.45 V, while the *i_corr_* value dropped to 8.9 μA·cm^−2^. Therefore, it can be inferred that incorporating the sodium silicate weakened the corrosion tendency and reduced the corrosion rate of the AZ31 Mg alloy. As the sodium silicate concentration was raised from 0.05 M to 0.10 M, the *E_corr_* value tended to be positive overall. In contrast, the *i_corr_* value exhibited a further decrease to 0.6 μA·cm^−2^, which indicated that, within the appropriate range of sodium silicate concentration, the ability to inhibit corrosion was improved. However, the icorr values increased slightly at a concentration of 0.15 M. In general, including the sodium silicate greatly enhanced the corrosion inhibition efficiency (*η* > 85%). Notably, the highest value of *η* (99.1%) was achieved by adding the 0.10 M sodium silicate to the corrosive solution.

**Figure 2:**
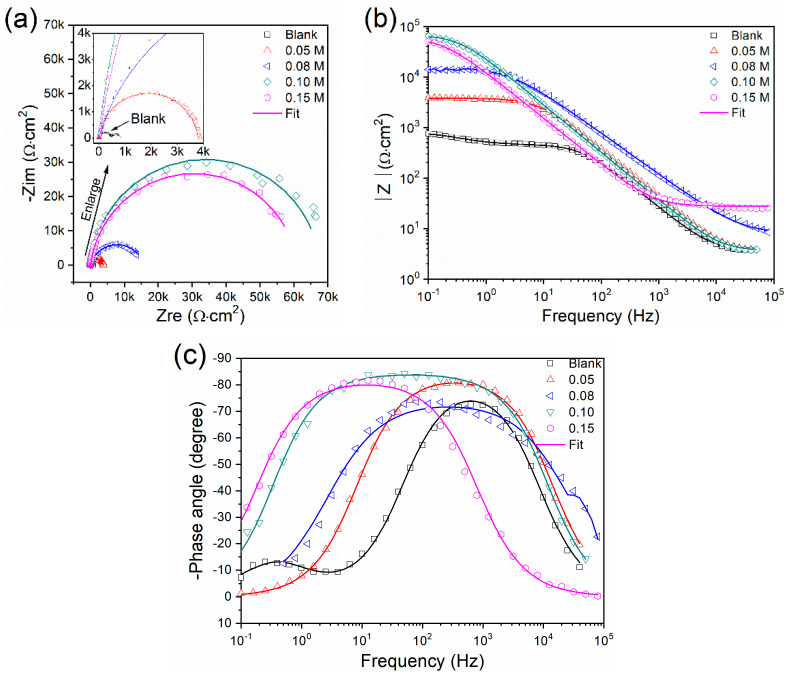
Electrochemical impedance spectroscopy of AZ31 Mg alloy measured in the solution containing 3.5 wt. % NaCl and x M sodium silicate (x = 0, 0.05, 0.08, 0.10, and 0.15): (**a**) Nyquist plot; (**b**) and (**c**): Bode plots.

The EIS results of the AZ31 Mg alloy measured in the solution containing the 3.5 wt. % NaCl and x M sodium silicate (x = 0, 0.05, 0.08, 0.10, and 0.15) are shown in Figure 2. The Nyquist plots of the AZ31 alloy in the blank concentration of the sodium silicate exhibited two capacitive loops, one at a high frequency and the other at a low frequency (Figure 2a). The high-frequency capacitive loop corresponded to the interface between the electrolyte and alloy, specifically referring to the electrical double layer and charge transfer resistance. The corrosion resistance is enhanced as the diameter of the capacitor loop increases. At the same time, the blank concentration group exhibited two capacitive rings, and only one capacitive loop was observed in the inhibitor groups. Notably, the size of the capacitive loop in the inhibitor group significantly surpassed that of the blank concentration group, indicating a substantial improvement in corrosion resistance [34].

The |Z| (impedance modulus) was commonly used to assess the erosion resistance of different samples [35]. A larger |Z| value in low frequency confirmed better corrosion inhibition performance. Figure 2b displayed that the inhibition effect was relatively poor when the concentration of the sodium silicate was less than 0.08 M.

Generally, a wider and higher phase angle (θ) in the middle frequency represented better properties of the protective layer, while that in low frequency was relative to the inner layer [36]. Therefore, the concentration of 0.10 M exhibited the best protection effect among all the concentrations, as shown in Figure 2c. In the blank concentration of sodium silicate, the Bode plot showed an additional phase angle at a low frequency, while the others did not. This was attributed to the addition of low-molecular sodium silicate from a compact film, which effectively inhibited the permeation of corrosive chloride ions, eliminating the phase angle at a low frequency [13].

**Figure 3:**
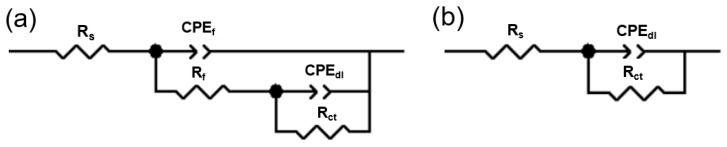
Equivalent circuit model: (**a**) blank concentration of sodium silicate; (**b**) x M sodium silicate (x = 0.05, 0.08, 0.10, and 0.15).

The circuit diagram and the corresponding data for fitting can be observed in Figure 3 and Table 3, respectively. *R*_s_ represents the solution resistance, and *R_f_* denotes the charge transfer resistance. A higher charge transfer resistance value represents a strong corrosion resistance. *CPE_dl_* and *CPE_f_* represent the constant phase element linked to the substrate–film interface’s low-frequency response and the electrolyte–film interface’s double-layer capacity [37,38,39]. *CPE_dl_* and *CPE_f_* represent the constant phase element linked to the substrate–film interface’s low-frequency response and the electrolyte–film interface’s double-layer capacity. *R_ct_* is the resistance of the film–electrolyte interface. Polarization resistance (*R_p_*) was commonly used to evaluate the anti-corrosion properties in electrochemical systems, and its calculation formula is provided by [40]

(7) $$R_{p}={R_{S}+R}_{ct}$$

The increasing concentration of sodium silicate caused a sharp increase in the *R_P_* value. The *R_p_* value of the 1.0 M sodium silicate addition was 67,845 Ω·cm^2^, while that of the blank concentration was 350 Ω·cm^2^. This suggests that the inhibition of corrosion in the AZ31 Mg alloy was successful. Moreover, the rise in the *R_p_* value was attributed to the increase in *R_ct_*_,_ which indicated that the difficulty regarding the transfer of charge and diffusion of the electrolyte was increased. As the adsorption of the sodium silicate increased the dispersion effect, the *CPE_dl_* value dropped with the increasing concentration of the sodium silicate. The above data indicate that the 1.0 M sodium silicate addition in the NaCl significantly enhanced the erosion resistance of the substrate by the adsorption on the surface.

### 3.2. Immersion Result

#### 3.2.1. Corrosion Rate

**Figure 4:**
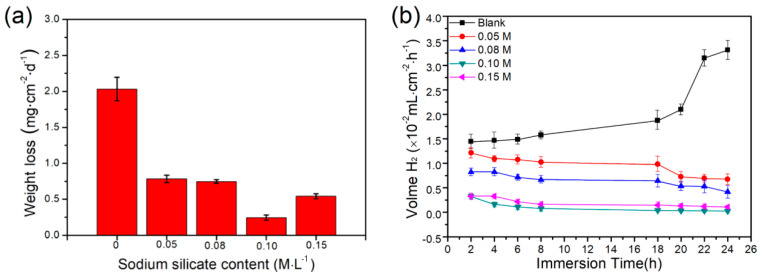
The corrosion rates of AZ31 Mg alloy after 24 h immersion in 3.5 wt. % NaCl solution containing x M sodium silicate (x = 0, 0.05, 0.08, 0.10, and 0.15): (**a**) weight loss and (**b**) hydrogen evolution.

Figure 4 shows the rate of hydrogen evolution volume and mass loss experienced by the AZ31 Mg alloy following a 24 h immersion in a NaCl solution with x M sodium silicate (x = 0, 0.05, 0.08, 0.10, and 0.15). The corrosion rates of *CR_I_*, *CR_W_*, and *CR_H_* are listed in Table 4. The weight loss rate of the blank concentration of sodium silicate was 2.032 mg·cm^−2^·d^−1^. When the concentration of the sodium silicate was 0.1 M, the AZ31 alloy exhibited the highest corrosion inhibition (the weight loss rate was 0.244 mg·cm^−2^·d^−1^) among these concentrations, as shown in Figure 4a. However, the addition of the 0.15 M concentration exhibited a slight decline in corrosion resistance, suggesting that an excessive concentration of sodium silicate can cause adsorption and desorption on the alloy surface. Meanwhile, the hydrogen evolution results in Figure 4b indicate that, during the whole immersion time, the corrosion rate of the blank concentration increased continuously, while the corrosion rate showed a decreasing trend with the addition of a corrosion inhibitor. Notably, the corrosion rates of the 0.10 M and 0.15 M concentrations of sodium silicate remained relatively low.

#### 3.2.2. Corrosion Morphology Characterization

**Figure 5:**
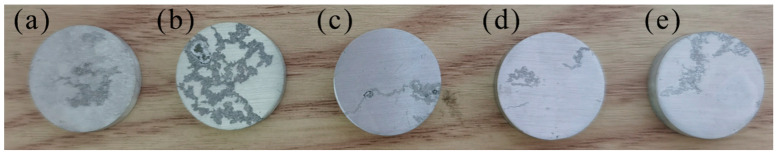
The macro-morphology of AZ31 Mg alloy after 24 h immersion in 3.5 wt. % NaCl solution containing x M sodium silicate and removal of corrosion products: (**a**) x = 0; (**b**) x = 0.05; (**c**) x = 0.08; (**d**) x = 0.10; (**e**) x = 0.15.

For a more intuitive characterization of the corrosion resistance, Figure 5 exhibits the morphology of the AZ31 alloy after 24 h immersion in a NaCl solution containing x M sodium silicate (x = 0, 0.05, 0.08, 0.10, and 0.15), as exhibited in Figure 5, and the AZ31 Mg alloy was severely corroded. When sodium silicate was added, the corrosion area on the surface decreased, and the uncorroded area retained the original metallic luster. This shows that the sodium silicate created a small protective layer on the alloy surface, effectively stopping the corrosion in the covered area. As the concentration increased, the corrosion area of the alloy was further reduced. The optimal corrosion resistance effect was attained at a 0.1 M sodium silicate concentration. The excess concentration of the sodium silicate (0.15 M) caused the corrosion resistance to slightly decrease. In summary, at a high concentration of sodium silicate (0.1 M), the sodium silicate can cover the alloy surface uniformly and play a role in corrosion inhibition. If the concentration of the sodium silicate further increases, silicates will present as polymers [41]. The presence of electrostatic repulsion between the polymer and alloy surface results in limited adsorption at a local level [42], thus resulting in decreased corrosion inhibition.

**Figure 6:**
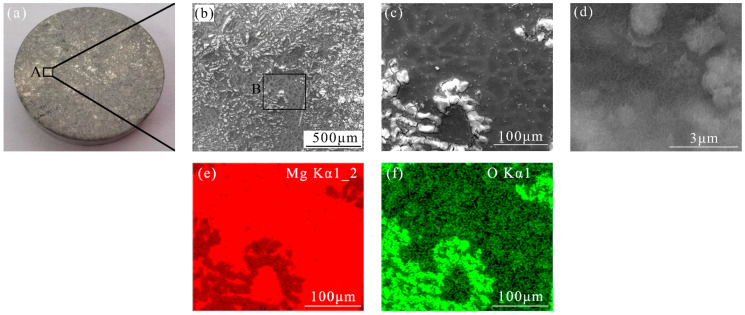
The SEM images and EDS results of AZ31 Mg alloy after 24 h immersion in a 3.5 wt. % NaCl solution: (**a**) macro picture; (**b**,**c**) SEM-enlarged images of Site A and Site B; (**d**) highly enlarged image of Site B; (**e**,**f**) EDS results of Site B.

Figure 6 illustrates the corrosion characteristics of the AZ31 magnesium alloy following a 24 h immersion in a NaCl solution without eliminating the presence of the corrosion byproducts. As shown in Figure 6a, the AZ31 Mg alloy has been severely eroded, and the corroded area occupied a large portion of the polished surface. The SEM-enlarged image of Site A indicates that a mass of corrosion products was deposited at all the corrosion sites. As shown in Figure 6c, the corrosion occurred mainly regarding the α-Mg phase [43], and the corrosion products layer was fractured. The EDS results in Figure 6e,f indicate that the corrosion products consisted primarily of Mg and O elements. Further enlarging the corrosion product area to Figure 6d, many loose porous structures were observed, and it was confirmed to be Mg hydroxide [44].

**Figure 7:**
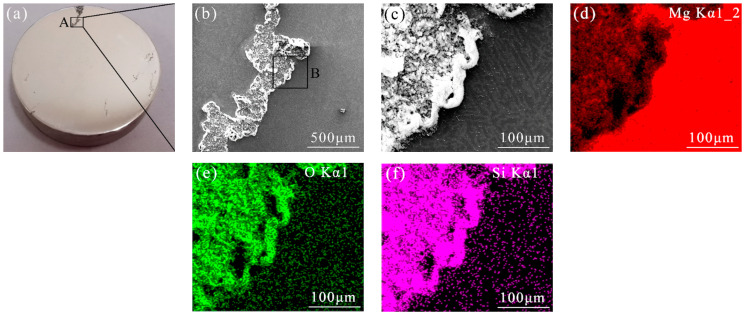
SEM images and corresponding EDS results of AZ31 Mg alloy after 24 h immersion in 3.5 wt. % NaCl solution containing 0.1 M sodium silicate: (**a**) macro picture; (**b**,**c**) SEM-enlarged images of Site A and Site B; (**d**–**f**) EDS results of Site B.

The effects of immersing the AZ31 Mg alloy in a NaCl solution with the addition of the 0.1 M sodium silicate for 24 h are depicted in Figure 7, and without removing the corrosion products. There was local corrosion on the polished surface, as shown in Figure 7a. The SEM image of Site A showed that continuous corrosion products were gathered on the sample’s corrosion area. As shown in Figure 7c, the corroded area was completely covered by the corrosion products, while the uncorroded area maintained good surface conditions. The EDS results in Figure 7d–f reveal that the predominant constituents of the white corrosion products were Mg, O, and Si, which fully indicated that sodium silicate was involved in forming the surface film. In addition, small amounts of Si and O elements were detected in the uncorroded area, indicating that the sodium silicate effectively adsorbed on the uncorroded area.

**Figure 8:**
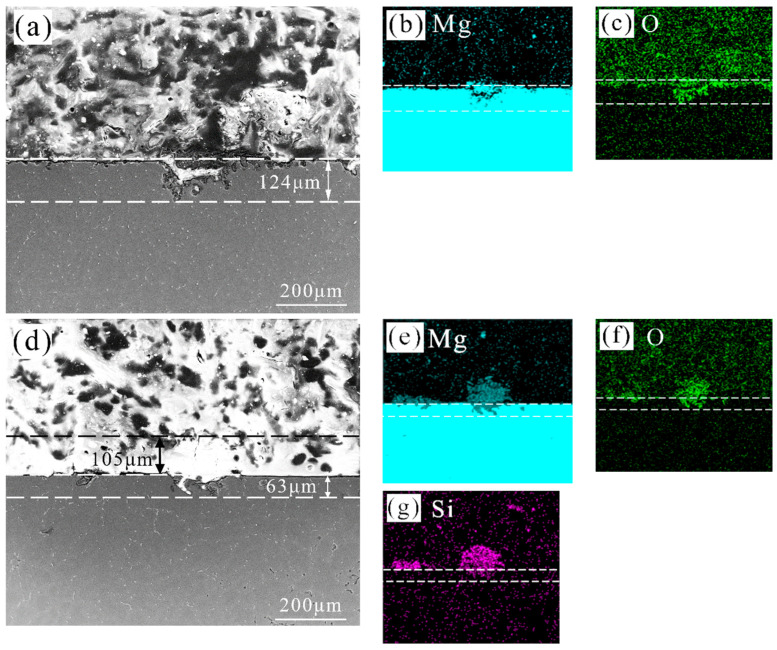
Cross-section images and EDS mappings of AZ31 Mg alloys after 24 h immersion in a 3.5 wt. % NaCl solution with and without 0.1 M sodium silicate addition: (**a**–**c**) without 0.1 M·sodium silicate addition; (**d**–**g**) with 0.1 M·L^−1^ sodium silicate addition.

Figure 8 displays the SEM profile images and the corresponding EDS results of the corroded samples. In Figure 8a–c, the corrosion depth of the local corroded area reached 124 μm relative to the blank concentration. Moreover, the area around the corrosion pit was also corrupted, which exhibited an uneven interface between the alloy and the mosaic powder. The EDS results showed that the O element was enriched in these areas, indicating the accumulation of magnesium hydroxide in the corrosion pits. Figure 8d–g are relative to the 0.1 M sodium silicate addition. The maximum depth of the local corrosion pits was 63 μm, which was about one-half that in the blank concentration. Moreover, the surrounding area around the corrosion pit did not exhibit obvious corrosion, and the interface of the sample and the mosaic powder was smooth. The EDS results displayed that the corrosion products consisting of Mg, Si, and O elements accumulated to a thickness of 105 μm in the local corrosion pit.

**Figure 9:**
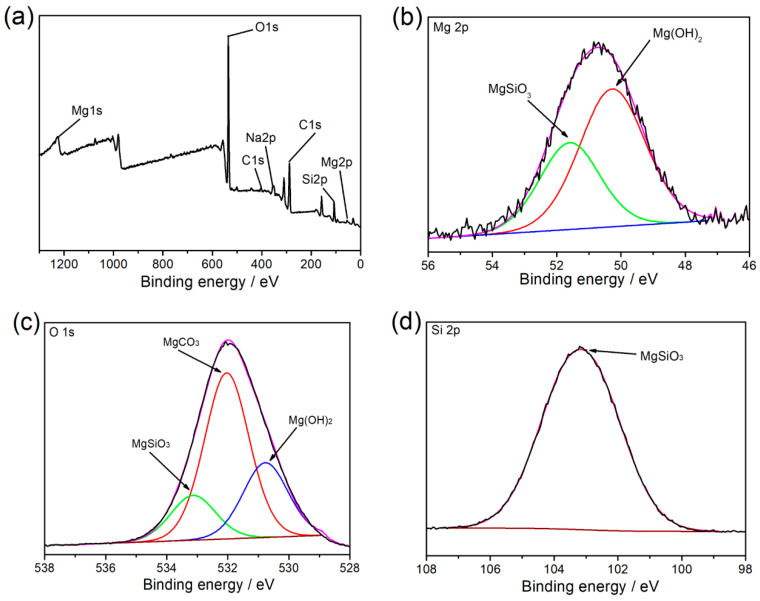
XPS spectra of the corrosion products on the surface of AZ31 Mg alloy immersed in a 3.5 wt. % NaCl solution with 0.1 M sodium silicate addition: (**a**) survey; (**b**) Mg2p spectrum; (**c**) O1s spectrum; (**d**) Si2p spectrum.

Figure 9 shows the XPS spectrum of Mg2p, O1s, and Si2p relative to the silicate film on the corroded surface. The composition of the surface elements within the silicate film encompassed Mg, O, C, Na, and Si. The Na element was the residual of the NaCl solution, while the C element came from the inorganic carbon in the air. In Figure 9b, the Mg element mainly existed in magnesium hydroxide and magnesium silicate. On the other hand, Figure 9c exhibits the peaks of MgO, C=O, and Si-O, which were relative to Mg(OH)_2_, MgCO_3,_ and MgSiO_3_. The Si2p peak in Figure 9d was characterized as silica and silicate at the binding energy of 103.2 eV. Therefore, the corrosion product accumulated on the corrosion crack was presumed to be MgSiO_3_ [30].

### 3.3. Adsorption Energy Calculation

**Figure 10:**
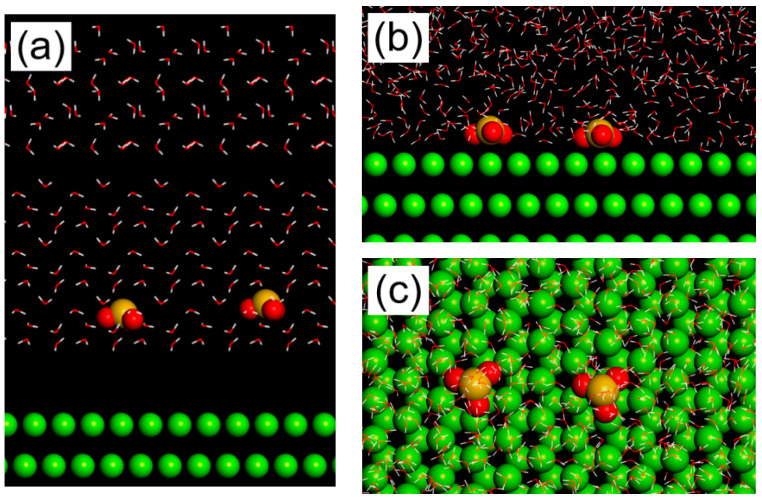
The initial side view and final top and side view of the adsorption configuration of AZ31 Mg alloy immersion in 3.5 wt. % NaCl solution with 0.1 M sodium silicate addition: (**a**) initial side view; (**b**) final side view; (**c**) final top view.

The stable adsorption configuration of the sodium silicate molecules on the Mg (0 0 0 1) surface is shown in Figure 10. As shown in the initial side view in Figure 10a, silicate ions and water molecules appeared on the Mg (0 0 0 1) surface. From the final top and side views (Figure 10b,c), the sodium silicate molecules were parallelly absorbed on the Mg (0 0 0 1) surface. The formulas [45] of the binding energy of the sodium silicate molecules’ absorption on the Mg (0 0 0 1) surface were

(8) $$E_{binding\;}=E_{total}-(E_{Mg+H_{2}O}+E_{{SiO}_{3}^{-}+H_{2}O})+E_{H_{2}O}$$

where $E_{total}$ is the total energy of the system, $E_{Mg+H_{2}O}$ is the total energy of the Mg crystal with all the H_2_O molecules, $E_{{SiO}_{3}^{-}+H_{2}O}$ denotes the total energy of $SiO_{3}^{-}$ and H_2_O, and $E_{H_{2}O}$ serves as the total energy of H_2_O. The calculation results indicate that the *E_binding_* of the sodium silicate molecules absorption on the Mg (0 0 0 1) surface was −8.091 kJ·mol^−1^. Previous studies [46,47] have revealed that chemical adsorption occurred when the binding energy was less than −40 kJ·mol^−1^, while physical adsorption occurred when the binding energy was higher than −20 kJ·mol^−1^. When the binding energy fell within the range of −40 to −20 kJ·mol^−1^, a phenomenon of physicochemical adsorption was observed. Hence, the AZ31 Mg alloy surface effectively captured the sodium silicate through physical adsorption, resulting in a binding energy of −8.091 kJ·mol^−1^.

## 4. Discussion

**Figure 11:**
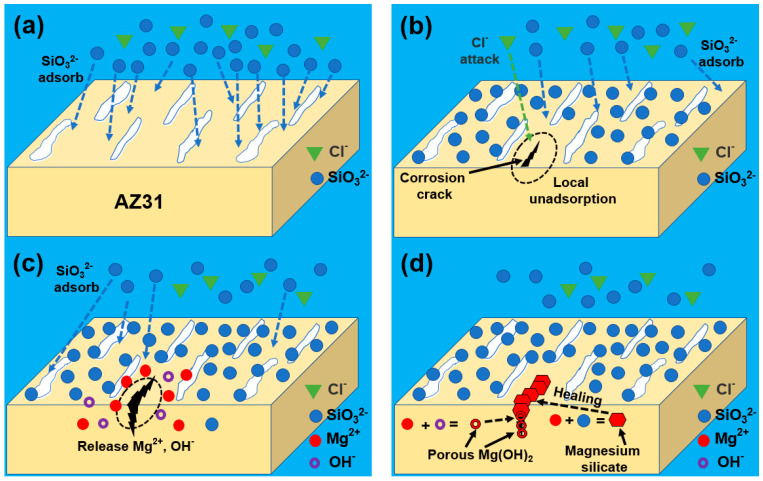
Schematic diagram (**a**–**d**) of the protective effect with sodium silicate film on AZ31 Mg alloy.

In this study, due to the addition of sodium silicate in the corroded solution, the SEM and EDS results (Figure 7) showed that the corrosion morphology was divided into the local corroded area and an uncorroded area and the Si element distributed in both regions. The distribution of the Si element in the local corroded area was quite a bit higher than that in the uncorroded area. The XPS results showed that the corrosion product mainly consisted of magnesium hydroxide, magnesium silicate, and silicon dioxide (Figure 9). The calculation of the adsorption energy of the sodium silicate showed that physical adsorption occurred on the surface of the Mg (0 0 0 1) surface (Figure 10). Focusing on the Si element enrichment in the corrosion area, the mechanisms of the protective effect of the sodium silicate film are illustrated by Figure 11, and the relative reactions that occurred in this process were provided as follows:

(9) $$Mg\;\to \;{Mg}^{2+}+2e^{-}$$

(10) $$2H_{2}O+2e^{-}\to H_{2}↑+\;2{OH}^{-}$$

(11) $${Mg}^{2+}+2{OH}^{-}\to Mg(OH{)}_{2}↓$$

(12) $${Mg}^{2+}+SiO_{3}^{2-}\to MgSiO_{3}↓$$

In the corrosive solution, as shown in Figure 11a, SiO_3_^2−^ and Cl^−^ ions appeared near the surface of the AZ31 Mg alloy. Silicate ions are adsorbed on the alloy surface to inhibit corrosion through physical adsorption, confirmed in the above electrochemical test and the calculation of the adsorption energy. Because of the locally uneven adsorption, as shown in Figure 11b, part of the matrix was exposed to the corrosive solution, and the action of the aggressive Cl^−^ resulted in the formation of a corrosion crack in this area. During this process, an anodic reaction occurred as in Equation (9). At the same time, this reaction occurred as in Equation (10). This is also evidenced by the corrosion morphology in Figure 7. As the crack expanded, large amounts of Mg^2+^ and OH^−^ were released in the solution, as shown in Figure 11c. A large amount of Mg^2+^ reacted with SiO_3_^2−^ to precipitate MgSiO_3_, as indicated in Equation (12), while some Mg^2+^ reacted with OH^−^ to precipitate Mg(OH)_2_ according to the reaction in Equation (11) (as shown in Figure 11d). The protective effect of the Mg(OH)_2_ was poor because of its loose and porous structure [48]. However, the high concentration of SiO_3_^2−^ caused the accumulation of large amounts of MgSiO_3_, which corresponded to the thickness of the corrosion products reaching 120 μm in Figure 8. This self-healing inhibitor effectively inhibited the corrosion, as mentioned in other research [49,50,51]. In conclusion, the sodium silicate inhibited the corrosion of the AZ31 Mg alloy through physical adsorption on the uncorroded surface, and the magnesium silicate was chemically precipitated in the corrosion crack [52,53,54].

## 5. Conclusions

(1) Electrochemical tests, surface analysis, and molecular dynamics simulations confirmed the effectiveness of sodium silicate on the corrosion of the AZ31 magnesium alloy. At a concentration of 0.1 M, the corrosion rate declined to 0.014 mm·y^−1^, resulting in the highest inhibition efficiency value of 99.1%. The corresponding polarization resistance was 67,845 Ω·cm^2^, while that of the blank concentration of sodium silicate was 350 Ω·cm^2^.
(2) Adding the 0.1 M sodium silicate caused a reduction in the weight loss rate of the AZ31 Mg alloy from 2.032 mg·cm^−2^·d^−1^ to 0.244 mg·cm^−2^·d^−1^. The excellent corrosion resistance was attributed to the precipitation of magnesium silicate within the corroded area with a thickness of 105 μm, and the sodium silicate was effectively adsorbed on the uncorroded area.
(3) Based on the molecular dynamics calculation, the sodium silicate was identified as a physical adsorption on the uncorroded area, and the binding energy was −8.09 kJ·mol^−1^. The protective mechanism of the sodium silicate inhibitor was explained as the synergistic action of the physical adsorption on the uncorroded area and chemical precipitation in the corroded area.

In conclusion, the present study shows that the 0.1 M sodium silicate is the most effective in protecting the AZ31 magnesium alloy in a NaCl medium to minimize corrosion and improve its corrosion resistance.

## Figures and Tables

**Table 1:** Analyzed compositions of the AZ31 Mg alloy (wt. %).

Al	Zn	Mn	Mg
3.06	0.86	0.14	Bal.

**Table 2:** Fitting parameters calculated according to the polarization curves in Figure 1.

Concentration (mol·L^−1^)	*E_corr_* (V_SCE_)	*i_corr_* (μA·cm^−2^)	*η* (%)	Corrosion Rate (mm·y^−1^)
0	−1.60	62.1	-	1.419
0.05	−1.45	8.9	85.7	0.203
0.08	−1.41	7.8	87.5	0.178
0.10	−1.44	0.6	99.1	0.014
0.15	−1.40	1.2	98.1	0.027

**Table 3:** Values of the AZ31 Mg alloys obtained from the EIS data.

Concentration (mol/L)	*R_s_* (Ω·cm^2^)	*CPE_dl_* (μΩ^−1^s^−n^cm^−2^)		*R_ct_* (Ω·cm^2^)	*CPE_f_* (μΩ^−1^s^−n^cm^−2^)		*R_f_* (Ω·cm^2^)	*R_p_* (Ω·cm^2^)
		*C*_1_	*n*_1_		*C*_2_	*n*_2_		
0	3.6	1.11 × 10^−5^	0.93	345.9	1.90 × 10^−3^	0.84	461.4	350
0.05	3.5	0.81 × 10^−5^	0.94	3788	-	-	-	3792
0.08	7.8	0.63 × 10^−5^	0.82	15,767	-	-	-	15,775
0.10	3.6	0.73 × 10^−5^	0.94	67,841	-	-	-	67,845
0.15	27.6	1.48 × 10^−5^	0.91	61,063	-	-	-	61,091

**Table 4:** The *CR_I_*, *CR_W_*, and *CR_H_* (the corrosion rates estimated from *i_corr_*, weight loss rate, and hydrogen evolution volume rate, respectively) of AZ31 alloy in the solution containing 3.5 wt. % NaCl and x M sodium silicate (x = 0, 0.05, 0.08, 0.10, and 0.15).

Concentration (mol·L^−1^)	CR_I_ (mm·y^−1^)	CR_W_ (mm·y^−1^)	CR_H_ (mm·y^−1^)
0	1.419	4.268	1.809
0.05	0.203	1.644	0.369
0.08	0.178	1.565	0.228
0.10	0.014	0.512	0.015
0.15	0.027	1.138	0.060

## Data Availability

The raw/processed data required to reproduce these findings cannot be shared at this time as they are also part of an ongoing study.
